# Cephalosporins’ Cross-Reactivity and the High Degree of Required Knowledge. Case Report and Review of the Literature

**DOI:** 10.3390/antibiotics9050209

**Published:** 2020-04-25

**Authors:** Stefano D’Errico, Paola Frati, Martina Zanon, Eleonora Valentinuz, Federico Manetti, Matteo Scopetti, Alessandro Santurro, Vittorio Fineschi

**Affiliations:** 1 Department of Medicine, Surgery and Health, University of Trieste, 34149 Trieste, Italy; sderrico@units.it (S.D.); martina.zanon@virgilio.it (M.Z.); eleonora.valentinuz@gmail.com (E.V.); 2 Department of Anatomical, Histological, Forensic and Orthopaedic Sciences, Sapienza University of Rome, 00161 Rome, Italy; paola.frati@uniroma1.it (P.F.); federico.manetti@uniroma1.it (F.M.); matteo.scopetti@uniroma1.it (M.S.); alessandro.santurro@uniroma1.it (A.S.)

**Keywords:** anaphylactic shock, ceftriaxone, cefepime, immunohistochemistry, liability, medical malpractice, R1 side-chain, R2 side-chain

## Abstract

Antibiotic cross-reactivity represents a phenomenon of considerable interest as well as antibiotic resistance. Immediate reactions to cephalosporins are reported in the literature with a prevalence of only 1–3% of the population, while anaphylactic reactions are rarely described (approximately 0.0001–0.1%) as well as fatalities. Allergic reaction to cephalosporins may occur because of sensitization to unique cephalosporin haptens or to determinants shared with penicillins. Cross-reactivity between cephalosporins represents, in fact, a well-known threatening event involving cephalosporins with similar or identical R1- or R2-side chains. The present report describes the case of a 79-year-old man who suddenly died after intramuscular administration of ceftriaxone. Serum dosage of mast cell tryptase from a femoral blood sample at 3 and 24 h detected values of 87.7μg/L and 93.5μg/L, respectively (cut-off value 44.3 μg/L); the serum-specific IgE for penicillins, amoxicillin, cephaclor and also for the most common allergens were also determined. A complete post-mortem examination was performed, including gross, histological and immunohistochemical examination, with an anti-tryptase antibody. The cause of death was identified as anaphylactic shock: past administrations of cefepime sensitized the subject to cephalosporins and a fatal cross-reactivity of ceftriaxone with cefepime occurred due to the identical seven-position side chain structure in both molecules. The reported case offers food for thought regarding the study of cross-reactivity and the need to clarify the predictability and preventability of the phenomenon in fatal events.

## 1. Introduction

Antibiotic allergy is defined as an immunologically mediated drug hypersensitivity reaction, either IgE- or non-IgE-mediated [1], and represents the most common cause of hypersensitivity (HSRs) and adverse drug reactions (ADRs) [2].

ADRs differ from adverse drug events (ADEs), as ADEs extend beyond ADRs to include injury resulting from medical errors [3]: ADEs are largely preventable and include medication errors, adverse drug reactions, allergic reactions and overdoses [4]. In hospitalized patients, antibiotic related-ADRs are associated with inferior clinical outcomes: microbiological resistance, restricted antibiotic use, adverse events, increased readmissions and excess mortality [5,6]. In the general population, antibiotics represent the commonest cause of life-threatening immune-mediated drug reactions that are considered off-target, where “off-target” is defined as being caused by different mechanisms of action rather than the intended primary pharmacologic mechanism [7]. Life-threatening drug reactions include anaphylaxis, organ-specific reactions and severe cutaneous adverse reactions (SCARs) [8].

Approximately 10% of the population is known to be antibiotic-allergic [9], so such reactions pose undeniable risk to patients, and addressing antibiotic allergy reactions currently represents a significant public health issue [10].

Penicillins, cephalosporins, monobactams and carbapenems (betalactam antibiotics, with a similar structure to a beta-lactam ring) are recognized as one of the most common causes of immediate (within one hour) and delayed (after 72 h) adverse drug reactions (ADRs), mediated by specific immunological mechanisms (IgE and non-IgE-mediated).

Immediate reactions to cephalosporins are reported in the literature with a prevalence of only 1–3% of the population: these reactions generally occur within one hour from administration, with symptoms represented mainly by urticarial, rhinitis and bronchospasm. Increasing serum IgE for cephalosporins is also observed: in most cases it is an idiopathic mechanism, without contraindications for future use of cephalosporins. On the other hand, anaphylactic shock is rarely described (approximately 0.0001–0.1%) as well as fatalities [11,12], in subjects with beta-lactam allergies. In particular, anaphylactic reactions following the administration of specific cephalosporins are reported and related to penicillins–cephalosporins’ or cephalosporins’ cross-reactivity [13].

Cross-reactivity between penicillins and first- and second-generation cephalosporins has been reported in 10% of penicillin-allergic patients. However, older studies may have overstated the cross-reactivity as the first cephalosporins contained traces of penicillins [14]. Cross-reactivity between penicillins and third-generation cephalosporins occurs in 2–3% of patients allergic to penicillins [15,16,17]. Cross-reactivity between cephalosporins can cause immune-mediated reactions in 1–3% of patients, even in the absence of a history of penicillin allergy [18].

As a consequence, prescription of antibiotics in subjects with known IgE-mediated hypersensitivity to beta-lactams is a big concern and the tolerability of an alternative cephalosporin is still debated. The risk for patients with a beta-lactams allergy is to receive suboptimal therapy, experience clinical failure, develop drug-resistant organisms and to have prolonged hospitalization and higher in-hospital mortality [19,20,21,22]. Common clinical practice suggests avoiding other beta-lactams in patients with a labeled beta-lactams allergy. Pre-treatment skin tests with alternative cephalosporins have also been proposed but doubts about validity still remain [23,24].

## 2. Case Report

A 79-year-old man suddenly died after intramuscular administration of ceftriaxone prescribed by a general practitioner (GP) because of cutaneous abscess. Dyspnea, cyanosis and cardiac arrest occurred immediately after administration and resuscitation maneuvers were unsuccessful. In the medical history, recurrent chronic obstructive pulmonary disease (COPD) exacerbations were recorded with antibiotic treatments in the last ten years, as described in Table 1. An episode of sudden lipothymia after administration of cefepime was also reported ten months before death and recorded as an allergic reaction in the electronic GP medical record. After death, medical malpractice of the GP was reported, and the day after death a complete post-mortem examination was ordered by the prosecutor.

Before the autopsy investigation, dosage of serum tryptase levels was performed on blood samples, sampled 3 h and 24 h after death and frozen at −20 °C. Serum tryptase levels were determined by EliA test based on fluorescence enzyme immunoassay (FEIA) (ImmunoCap Tryptase, Phadia 250: Thermo Fisher Scientific, Phadia AB, Uppsala Sweden). Serum dosage of β-tryptase from femoral blood detected values of 87.7 μg/L and 93.5 μg/L respectively (cut-off value 44.3 μg/L [25]). Serum-specific IgE for penicillins, amoxicillin, cephaclor and also for the most common allergens were also determined on the cadaveric blood samples by EliA test, based on fluorescence enzyme immunoassay (FEIA) (ImmunoCAP Allergen Components, Phadia 250: Thermo Fisher Scientific, Phadia AB, Uppsala Sweden), as reported in Table 2.

The external examination was normal except for a cutaneous abscess localized in the right gluteus. The internal examination was unremarkable except for heavy lungs and abundant reddish-colored foam on the main bronchi. The other organs did not show any specific pathological alterations except for cerebral edema.

All tissue specimens were fixed in formalin and embedded in paraffin, and a routine microscopic histopathological study was performed using hematoxylin-eosin (H&E). Acute polivisceral stasis, mild cerebral edema and interstitial myocardial edema were observed. Chronic and acute pulmonary emphysema, as well as massive pulmonary edema, were observed in all samples.

**Figure 1:**
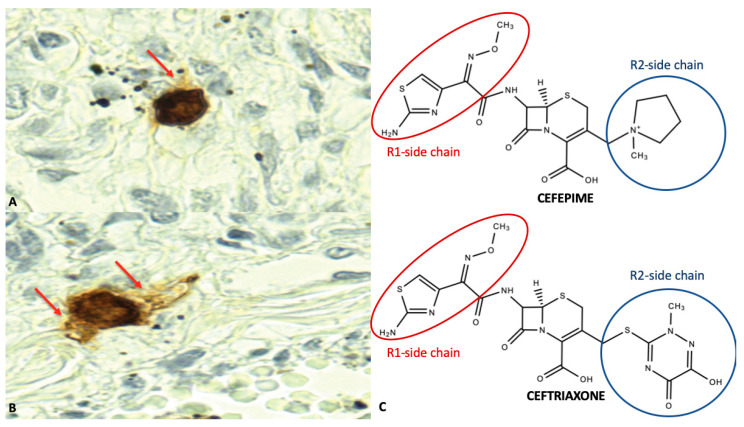
(**A**,**B**) Immunohistochemical investigation using antibodies anti-tryptase for lung sections: degranulating mast cells with tryptase-positive material outside (red arrows) were observed; (**C**) Chemical structures of cephalosporins: cross-reactivity between ceftriaxone and cefepime, sharing a similar seven-position side chain (R1).

An immunohistochemical investigation to assess the mast-cell population was performed using antibodies anti-tryptase for lung sections. Enzyme pre-treatment with proteinase K (0.01% at 37 °C) was necessary to facilitate antigen retrieval and to increase membrane permeability to antibodies. The primary antibody anti-tryptase (Agilent-Dako, Santa Clara, CA, USA) was applied at a 1:100 ratio and incubated overnight at 4 °C. The positive reaction was visualized by 3-amino-9ethyl-carbazole (AEC) (Sigma-Aldrich Merck, Darmstadt, Germany). The sections were counterstained with Mayer’s hematoxylin and mounted in Aquatex (Merck Pharma, Darmstadt, Germany). A quantitative analysis was performed in each histological section, with 10 observations in different fields per slide equivalent to 70 observations. The positive mast-cell count to the tryptase reaction was made at a magnification of 10× using a light microscope coupled to a high-resolution color video camera: a pulmonary area of 100 mm^2^ was analyzed. Pulmonary mast cells were identified and quantified, and a great number of degranulating mast cells with tryptase-positive material outside were observed (Figure 1). Data resulting from quantitative analysis recorded a numerical increase in pulmonary mast cells (average mast-cell count 11,951/100 mm^2^) compared with a control group represented by traumatic deaths (average mast-cell count 3557/100 mm^2^).

Toxicological analysis on urine and blood specimens was performed using gas chromatography-mass spectrometry (GC-MS) and resulted negative.

A fatal anaphylactic shock was recorded as the cause of death: it was supposed that past administration of cefepime with lipothymia sensitized the subject to cephalosporins and a fatal cross-reactivity between ceftriaxone and cefepime, sharing a similar seven-position side chain, occurred. The court excluded medical malpractice of the GP because of the high degree of knowledge required to discriminate between cephalosporins with similar or identical R-side chains and the difficulty to predict cross-reactivity before administration beyond reasonable doubt.

## 3. Discussion

Cephalosporins represent one of the most commonly prescribed classes of antibiotics for pulmonary, skin and soft tissue infections due to their broad spectrum of activity and low toxicity profile. Anaphylactic reactions from cephalosporins are extremely rare and the incidence of allergy is estimated to be 1–3% of the general population [26,27]. A French report in 2005 described a 27% prevalence of severe allergic reactions to cephalosporins among all cases involving β-lactams [28].

Cross-reactivity between cephalosporins and penicillins has been widely investigated in the past and the safety of cephalosporins’ prescription in patients primarily sensitized to penicillins has been deeply analyzed [29]. Recent data suggest that 1–4% of patients with a history of penicillin allergy have a true cephalosporin allergy. Skin manifestations (1–5%), fever (0.5–0.9%), eosinophilia (2–10%) and anaphylaxis (<0.1%) are the most commonly reported clinical signs in case of cephalosporin allergies [26]. In other studies, the incidence of anaphylaxis to cephalosporins is estimated as 0.0001–0.1% [30].

Cross-reactivity between cephalosporins has been less studied than the reaction to cephalosporins in patients with primary sensitization to penicillins. While the chemical structure of the allergenic determinants of penicillins is known, the allergenic determinants of cephalosporins have not been completely elucidated. Thus, standardized diagnostic tests for cephalosporin allergy are not available [31].

Some studies have demonstrated that cephalosporins generate unique structures capable of provoking an IgE-mediated immunologic response [32]. Cephalosporins consist of a six-membered dihydrothiazine ring fused to a core, four-membered cyclic amide beta-lactam ring with two side chains, R1 and R2. The chemical structure of cephalosporins differs significantly in side chains bound to the R sites. These differences explain variation in the spectrum of activity and duration of action of individual cephalosporins grouped into five generations. Both the R1- and the R2-side chains may contribute to the configuration of different epitopes [33]. When IgE antibodies react with cephalosporins, they may recognize a portion of a side chain, the methylene group, full-side chains, a combination of a side chain and part of the beta-lactam ring and even the entire cephalosporin molecule [14,34,35,36].

**Scheme 1:**
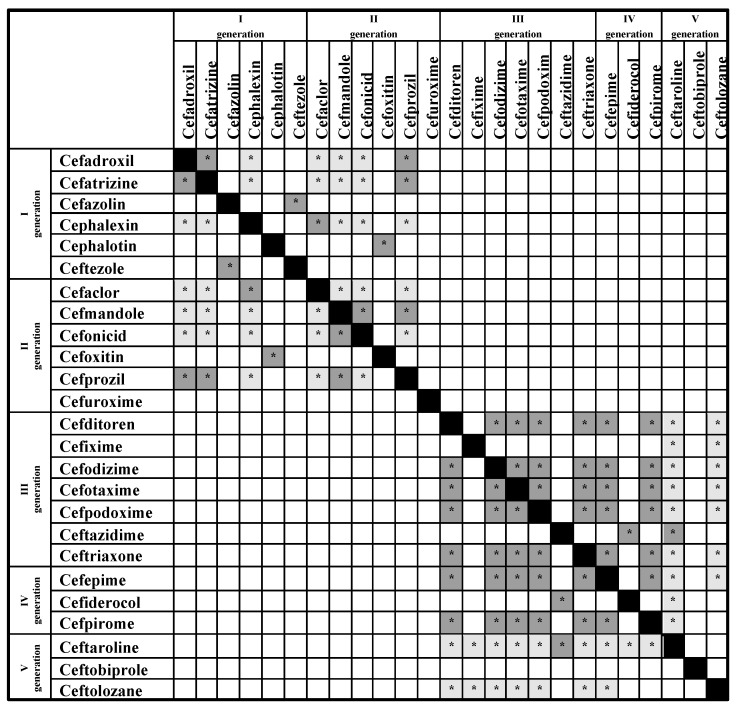
Cephalosporins sharing identical (dark grey) or similar (light grey) R1-side chains.

Wide evidence exists about aminobenzyl R1-side chains of cephalosporins’ involvement in allergic reactions, so that it is often considered the only allergenic structure of cephalosporins. Cross-reactivity between cephalosporins represents, in fact, a well-known threatening event involving cephalosporins with similar or identical R1-side chains [37] (Scheme 1). Cross-reactivity between ceftriaxone, cefuroxime, cefotaxime and cefozidime has been widely investigated, because of identical (ceftriaxone and cefotaxime) or similar (ceftazidime) R1-side chains. In particular, identical R1-side chains were demonstrated in cases of cross-reactivity between ceftriaxone and cefotaxime [38]. In other cases, even slight differences in the R1 side chain should make IgE-mediated reactions unexpected [39]: Pichichero observed cephalosporins that share a similar seven-position or three-position side chain are more likely to cross-react with each other [11].

Additionally, R2-side chains’ involvement cannot be excluded, and nor can a combined effect of R2-side chains with the R1 methoxymino (Table 3). The rupture of the dihydrothiazine ring occurring during cephalosporin degradation leads to the expulsion of the R2 group while the R1 group remains intact [37,40,41,42]. Additionally, immediate hypersensitivity was reported between cefoperazone and cefamandole sharing an identical R2-side chain [43].

Lastly, in other cases, a selective hypersensitivity to individual cephalosporins without cross-reactivity has been reported, suggesting that the entire cephalosporin molecule could be involved in reactions [44].

Hypersensitivity reactions to cephalosporins generally occur within 24 h of drug exposure and consist of cutaneous rashes [26]. Despite the rarity of fatal events, worries regarding prescriptions and litigation surfaced early [38,45,46]. In fact, the evaluation of patients with primary hypersensitivity to β-lactams and cephalosporins is often not sufficiently addressed and the risk of litigation is high [47,48,49,50].

In patients with a true IgE-mediated reaction to cephalosporin, substituting cephalosporins to avoid allergy is mandatory and knowledge of the similarities and differences between R1 groups and R2 groups must be necessarily requested to avoid threatening consequences for patients. In particular, considering side-chain groups for the prescription of cephalosporins in a subject with previous IgE-mediated hypersensitivity to these beta-lactams is considered of paramount importance [51]: in these cases, cephalosporins with dissimilar side chains should be prescribed.

Despite the valuable results of skin tests, used to diagnose immediate hypersensitivity to cephalosporins, the limitations must be clearly considered [52]. The rate of positive skin tests to cephalosporins in patients with a history of confirmed hypersensitivity reactions varies sensitively from 0.3% to 69.7% [46,53]. A negative predictive value of skin tests of 82% was estimated [54]. Additionally, routine screening with an intradermal test with cephalosporin prior to administration does not predict immediate hypersensitivity and it can lead to false drug allergy labelling [55,56].

Before the administration of cephalosporin in a patient with known hypersensitivity to cephalosporins, induction of drug tolerance or graded challenge procedures may be taken in account [31].

## 4. Conclusions

The reported case enhances the value of a detailed history in cases of suspected cephalosporin allergy. Symptoms, the class of cephalosporin involved in primary hypersensitivity, the R-group side chain, the route of administration and previous reactions to penicillins or other cephalosporins should be investigated. The implementation of reporting systems and further research are desirable to reduce the incidence of adverse drug reaction and improve administration safety. Such an effort could allow the individualization of future antibiotic therapies, that should be tailored on the basis of potential cross-reactions [57,58].

In the reported case, the court assigned sharing of identical or similar R1- and R2-side chains of cephalosporins in the sphere of the “high degree” of knowledge not requirable to general practitioners. The difficulty to predict cross-reactivity before administration beyond a reasonable doubt acted in favor of the GP.

## Figures and Tables

**Table 1:** Schedule of antibiotics administration over a wide range of time (ten years).

DOP ^1^	ROA ^2^	Antibiotic
05.02.2003	i.m. 1 fl 1 gr	Ceftazidime
15.09.2006	cpr 500 mg	Ciprofloxacin
03.11.2006	i.m. 1 gr	Ceftriaxone
24.11.2006	os 875 mg + 125 mg	Amoxicillin + Clavulanic acid
05.05.2008	cps 400 mg	Ceftibuten
09.06.2008	i.m. 1 fl 2 gr	Piperacillin + Tazobactam
09.06.2008	cpr 500 mg	Levofloxacin
15.12.2010	im 1 fl 1 gr	Ceftriaxone
17.12.2010	im 1 fl 1 gr	Ceftriaxone
30.12.2010	cpr 750 mg	Ciprofloxacin
31.01.2011	cpr 750 mg	Ciprofloxacin
23.02.2011	im 1 fl 1 gr	Cefepime
04.04.2011	cpr 875 mg	Amoxicillin + Clavulanic acid
10.11.2011	cpr 750 mg	Ciprofloxacin
09.02.2012	cpr riv 500 mg	Ciprofloxacin
20.02.2012	cpr 500 mg	Levofloxacin
06.04.2012	i.m. 1 fl 1 gr	Cefepime *
06.04.2012	cpr 750 mg	Ciprofloxacin
19.04.2012	cpr 875 mg	Amoxicillin + Clavulanic acid
19.04.2012	cpr 500 mg	Levofloxacin
06.11.2012	i.m. 1 fl 2 gr	Piperacillin + Tazobactam
06.11.2012	cpr 750 mg	Ciprofloxacin
12.11.2012	cpr 750 mg	Ciprofloxacin
06.12.2012	cpr 750 mg	Ciprofloxacin
03.01.2013	i.m. 1 fl 2 gr	Piperacillin + Tazobactam
03.01.2013	cpr 750 mg	Ciprofloxacin
18.02.2013	i.m. 1 fl 1 gr	Ceftriaxone
18.02.2013	cpr 875 mg	Amoxicillin + Clavulanic acid

* In these circumstances, a lipothymia-like episode occurred immediately after administration. ^1^ DOP: date of prescription. ^2^ ROA: route of administration.

**Table 2:** Fluorescence enzyme immunoassay (FEIA) of blood samples collected 3 h and 24 h after death for detection of specific IgE for penicillins, ampicillin, amoxicillin, cephaclor and for the most common allergens.

Allergens	Blood—3 h After Death *	Blood—24 h After Death *
c1 (Penicillin G)	1.08	0.26
c2 (Penicillin V)	**3.47**	**1.57**
c5 (Ampicillin)	1.33	0.45
c6 (Amoxicillin)	1.26	0.26
c7 (Cefaclor)	1.36	0.53
g6 (Timothy grass-*Phleum pratense*)	1.75	0.55
t9 (Olive-*Olea europaea*)	1.20	0.27
t23 (Cypress-*Cupressus sempervirens*)	1.28	0.28
f1 (Egg)	1.97	0.83
f2 (Milk)	1.67	0.46
d1 (*Dermatophagoides pteronyssinus*)	1.31	0.33

* kUA/mL.

**Table 3:** Cephalosporins sharing identical or similar R2-side chains.

	Exact R2-Side Chains	Similar R2-Side Chains
Cefazolin		Ceftezole
Cefepime		Cefiderocol
Cefiderocol		Cefepime
Cefixime	Cefdinir	
Cefmandole	Cefoperazone, Cephapirin	Cefuroxime
Cefonicid		Cefmandole, Cefoperazone, Cefotetan
Cefotaxime	Cephalotin, Cephapirin	Cefuroxime
Cefoxitin	Cefuroxime	Cefotaxime, Cefoxitin, Cephapirin
Cefpirone		Ceftazidime
Ceftezole		Cefazolin
Cefuroxime	Cefoxitin	Cefotaxime, Cephalotin, Cephapirin
Cephalotin	Cephapirin	Cefoxitin, Cefuroxime
